# Underwater fiber laser removal of synthetically cultured *Caulobacter crescentus* biofilms on aluminium using response surface methodology

**DOI:** 10.1038/s41598-025-11455-3

**Published:** 2025-07-15

**Authors:** Hawa Ringkai, Khairul Fikri Tamrin, Ngieng Ngui Sing, Abdullah Yassin, Awang Ahmad Sallehin Awang Husaini, Jamil Musel

**Affiliations:** 1 Department of Mechanical and Manufacturing Engineering, Faculty of Engineering, Universiti Malaysia Sarawak (UNIMAS), 94300 Kota Samarahan, Sarawak Malaysia; 2 Faculty of Resource Science and Technology, Universiti Malaysia Sarawak (UNIMAS), 94300 Kota Samarahan, Sarawak Malaysia; 3 Department of Fisheries Malaysia, Fisheries Research Institute Sarawak, 93744 Kuching, Sarawak Malaysia

**Keywords:** Laser materials processing, Laser optical measurement, Biofilm, Biofouling, Freshwater, Microbiology, Environmental sciences, Engineering, Materials science, Optics and photonics

## Abstract

Biofouling can be considered the accumulation of microorganisms and other organisms on submerged surfaces. It poses operational and economic challenges across marine, industrial, and freshwater systems. Despite its relevance, freshwater biofilm removal remains understudied compared to marine biofouling. This study evaluates the use of fiber laser treatment to remove synthetically cultured *Caulobacter crescentus* biofilms formed on aluminium substrates submerged in a nutrient-rich microbiological growth medium. A response surface methodology was applied to optimize three laser processing parameters: laser power, laser speed, and repetition loops. The aim was to maximize biofilm removal efficiency while minimizing substrate damage. Results revealed that the number of repetition loops was the most significant factor, as multiple repetition loops compensated for energy losses caused by water absorption and the high thermal conductivity of aluminium. While increased laser power enhanced removal, its individual impact was less pronounced, and laser speed had minimal effect due to rapid thermal dissipation and water interference. A distinct white line, visible both macroscopically and microscopically, appeared in some laser-treated areas. Its origin is hypothesized to involve oxidation or material ablation, warranting further analysis. Response surface analysis demonstrated a non-linear relationship between biofilm removal width and both laser power and repetition loops, with peak efficiency observed at intermediate laser power levels. The observed curvature is attributed to the interplay of water turbulence, microbubble formation, and localized heat transfer dynamics. These findings highlight the complex nature of laser–biofilm interactions in submerged environments. This study contributes to the optimization of non-contact laser cleaning techniques for submerged applications and offers practical insights for industries such as water treatment, biomedical device maintenance, and antifouling surface design.

**Supplementary Information:**

The online version contains supplementary material available at 10.1038/s41598-025-11455-3.

## Introduction

Freshwater biofouling is a serious environmental problem which leads to bigger issues for submerged surfaces and machinery. Biofouling is the result of the adhesion of bacteria to surfaces, which plays critical roles negatively in the environment and industry. Freshwater biofouling can affect water-powered energy generation systems, boat hulls, harbours and piers as well as underwater engineering installations. Fishing and fish farming are also affected, with mesh cages and trawls harbouring fouling organisms. Microfouling, i.e., biofilm formation on surfaces, can have an economic impact and requires costly maintenance (e.g., biofouling adds considerable weight to buoyancy and anchoring systems), where the performance, accuracy and reliability are negatively impacted by the growth of fouling organisms.

There are over one thousand species of organisms (bacteria, viruses, protozoa, fungi, and algae) that cause biofouling in freshwater environments. The development of biofouling in these environments typically follows a regular sequence of events: (a) development of a conditioning film, (b) microfouling, and then (c) macrofouling^1^. The development of a conditioning film usually occurs within seconds and provides the linking layer between the fouling organism and the substrate surface^2^. Bacteria and other microorganisms in the vicinity of the substrate initially attach to the conditioning film via electrostatic forces, hydrogen bonds, and van der Waals forces^3^. This eventually alters porosity, density, water content, charge, sorption properties, hydrophobicity, and the mechanical stability of the biofilm^4^. Microbial biofilms become a nuisance when their development exceeds a tolerance threshold that allows them to damage materials and degrade component performance^4^. Biofilms also alter the release of biocides from antifouling coatings^2^.

The freshwater bacterium *C. crescentus* is usually the first organism to colonize any watery surface, from boat hulls, to water pipes, to submerged machinery. It attaches to solid surfaces through an adhesive holdfast located at the tip of its polar stalk, a thin cylindrical extension of the cell membrane. Removing it requires an average force around 70 N/mm^2^ to detach it from a surface^5^, a grip about seven times stronger than that of a gecko’s footpad. In other words, *C. crescentus* holdfast covering 1 cm^2^ would have the potential to resist a force of 700 kg on a wet surface. Meanwhile, some commercial ‘super’ glue gives up at about 25 N/mm^2^. Hence, *C. crescentus* can resist high-pressure water jets with ease and is notoriously difficult to remove.

The most common methods used for biofouling removal are dry-docking cleaning, chemical antifouling paint, and periodic underwater cleaning. Dry-dock cleaning is not economically feasible, while traditional chemical antifouling is less environmentally friendly. Mechanical removal of biofouling using brushes, scrapers, and other abrasive means can be done either in dry conditions or underwater at the expense of long operation cycles and high labour intensity. These mechanical methods can damage welds, rivets, and protrusions on structures, thereby compromising their mechanical integrity. Non-contact removal of microfouling and macrofouling using high-pressure water jets (sometimes containing abrasives) may cause irreparable damage to the underlying coating which in turn leads to accelerated corrosion when protective coating on the surface is removed. Not only is it expensive to routinely clean surfaces, but long-term repair and maintenance may also require significant investment.

One of the promising solutions in addressing these issues is laser radiation. This approach is highly regarded as a non-contact green technology since only electromagnetic waves are involved without the need for chemical use. It offers several key advantages over conventional dry or air-based methods, particularly in sensitive surface treatments. The water layer helps reduce surface oxidation by limiting exposure to air, improves thermal control by confining heat within a narrow zone, and assists in removing debris more effectively during ablation^6^. These features make it well-suited for the precise removal of biofilms in submerged environments. There is very limited literature on laser cleaning of micro-biofouled surfaces in oceanic applications. For instance, the efficacy of the laser radiation has been evident in the removal of seawater micro-biofoulings from AH36 steel substrate^7^ and Al-Mg-Si series aluminium alloy^8,9^. The application of fiber laser technology for freshwater biofouling removal remains largely unexplored, as most existing studies focus on marine environments. Its effectiveness in biofilm removal particularly with fiber lasers requires further investigation. Additionally, key laser parameters, such as laser power, laser speed, and repetition loops have not been optimized for efficient biofilm removal. Most biofilm studies also rely on general biofouling organisms, whereas *C. crescentus*, a novel freshwater bacterium, provides a controlled biological model to examine biofilm formation and laser-material interactions. A deeper understanding of these interactions is essential for developing fiber laser-based biofouling removal strategies while minimizing substrate damage. Hence, the primary objective of this study is to investigate the effects of fiber laser parameters on the removal efficiency of *C. crescentus* biofilm formed on aluminium substrates in a submerged environment, optimizing laser settings to achieve effective biofilm removal while minimizing substrate damage.

## Methodology

### Revival of *C. crescentus* BAA-2331 from powder form for working culture preparation

The preparation of *C. crescentus* BAA-2331 working culture from its powdered form was conducted under aseptic conditions to ensure successful revival and bacterial viability^10^.

#### Media preparation and sterilization

**Fig. 1:**
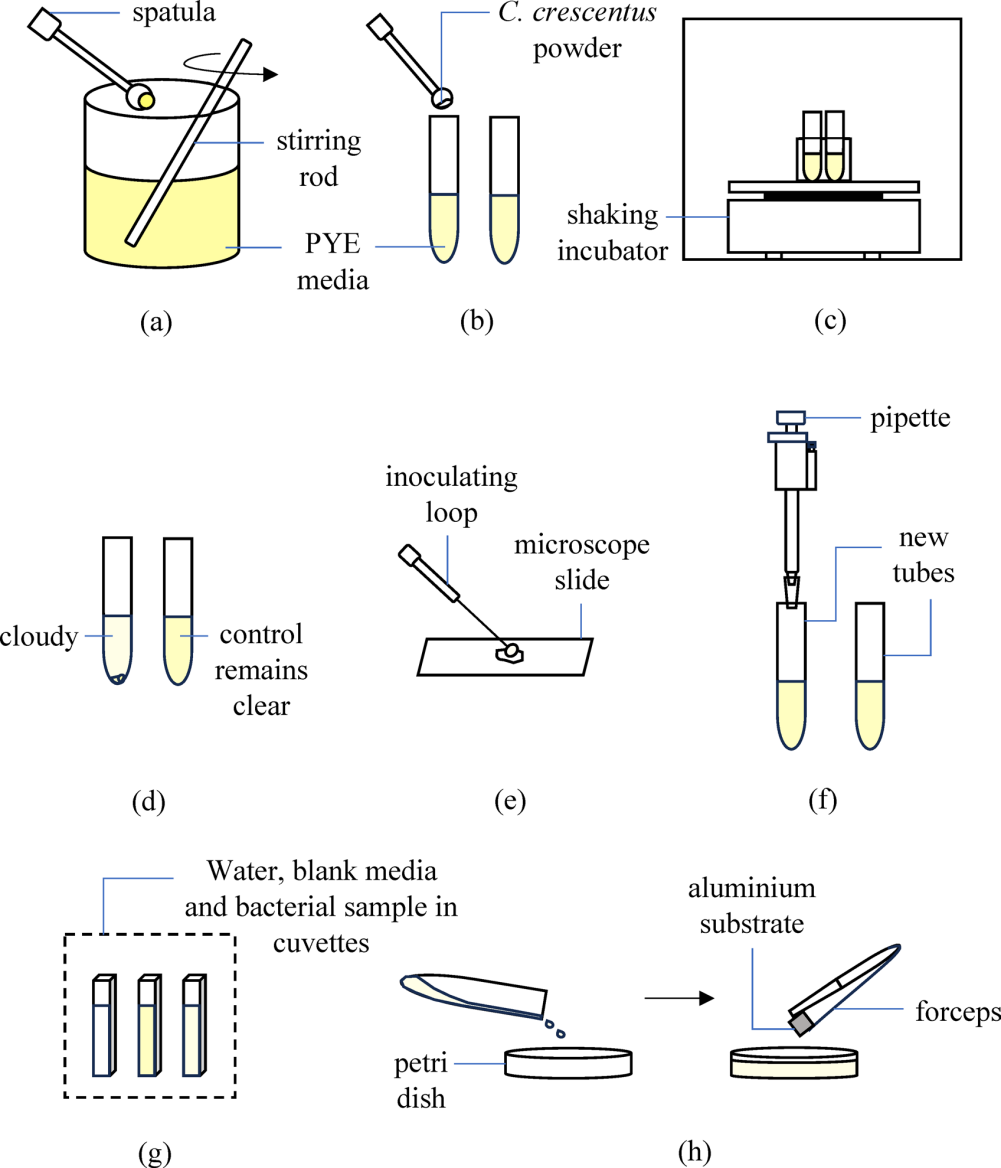
The step-by-step diagram of bacteria revival and biofilm formation setup.

To revive *C. crescentus* which was originally in powdered form, the growth medium, peptone-yeast extract (PYE), was prepared by dissolving 1.0 g peptone, 0.5 g yeast extract, and 0.1 g MgSO₄·7 H₂O in 500 mL tap water. The solution was mixed thoroughly until a clear yellowish liquid formed (Fig. 1 (a)) and sterilized using an autoclave machine (HVE-50, Hirayama) at 121 °C for 1.5 h. After sterilization, the liquid medium was left to cool.

#### Aseptic handling and bacterial inoculation

To prevent contamination, all necessary tools, including a Bunsen burner, inoculation loop, forceps, gloves, alcohol spray, and other aseptic handling equipment, were prepared. Before each handling step, disinfectant alcohol was sprayed onto the gloves, and the workspace was sanitized. The Bunsen burner was ignited to create a sterile working environment, and the openings of bottles, tubes, and tool contact areas were briefly exposed to the flame for sterilization.

Using an aseptic technique, approximately 20 mL of PYE media was distributed into two labelled tubes designated for experimental and control conditions. A sterile spatula was used to transfer a small amount of freeze-dried *C. crescentus* strain CB2A (ATCC BAA-2331)^11^ into the experimental tube (Fig. 1b), which was then securely sealed with parafilm tape.

#### Incubation and growth monitoring

Both the experimental and control tubes were placed in a shaking incubator (NB-205 L, N-Biotek) at 35 °C with orbital agitation to promote bacterial growth (Fig. 1c). The cultures were incubated overnight. After incubation, all apparatus and tools were cleaned, and the freeze-dried *C. crescentus* powder was returned to a − 80 °C storage freezer for future use. The next day, bacterial growth was assessed by visually comparing the experimental and control tubes. Successful revival was indicated by a cloudy or milky appearance in the experimental tube, while the control tube remained clear (Fig. 1d). Further confirmation of *C. crescentus* morphology was performed using a microscope.

### Microscopic confirmation of *C. crescentus*

#### Slide preparation and staining

A bacterial sample was prepared for microscopic examination to confirm the presence and morphology of *C. crescentus*. The working area was first disinfected with an alcohol spray to maintain aseptic conditions. A clean, dry microscope slide was placed on the table. A pipette (Finnpipette F2, Thermo Scientific) was used to place a tiny drop of distilled water at the center of the slide. Using a sterile metal inoculating loop, a small amount of bacterial suspension was collected from the experimental tube and spread onto the drop of distilled water (Fig. 1e). The slide was briefly passed over a flame to heat-fix the bacteria, ensuring proper adhesion. Safranin Gram stain was applied to the bacterial smear and left for approximately 30 s to allow absorption. Excess stain was rinsed off with tap water, and the slide was gently dried.

#### Microscopic examination

**Fig. 2:**
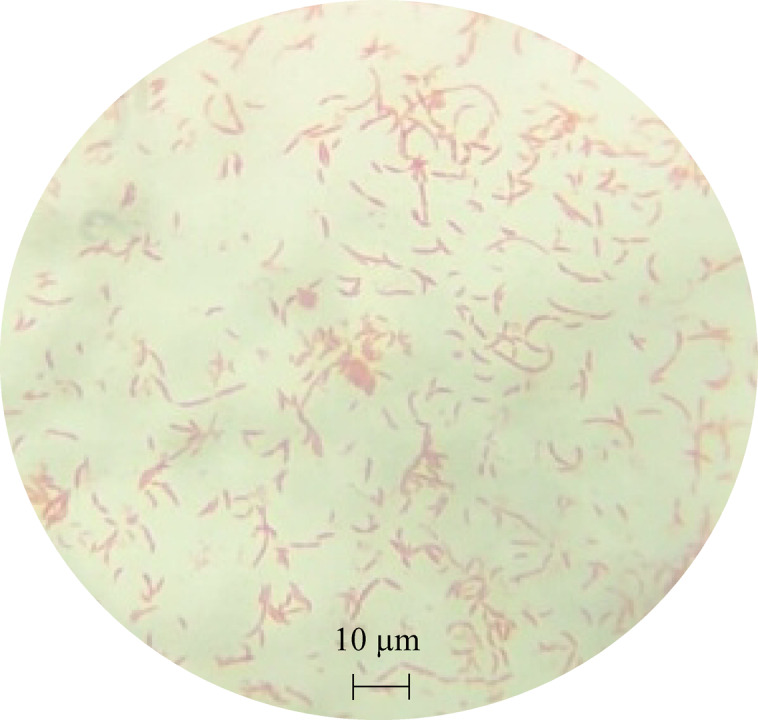
The morphology of the revived *C. crescentus*.

The sample was examined under a microscope (B-150, Optika) using a 100× oil immersion lens, which enhanced resolution and minimized light refraction. The bacterial morphology was confirmed as *C. crescentus*, exhibiting its characteristic crescent-shaped, rod-like structure (Fig. 2).

### Dilution of *C. crescentus* working culture and glycerol stock preparation

#### Preparation of diluted working culture

Two new tubes with proper labels were prepared, one for the diluted working culture and another for the new control. The workspace was disinfected, and proper aseptic techniques were maintained throughout the procedure.

For the dilution process, 30 mL of fresh PYE media was transferred into a sterile experimental tube. Then, 5 mL of bacterial suspension from the main culture tube was added to ensure a controlled bacterial concentration (Fig. 1f). The tube was sealed with parafilm, along with the control and main culture tubes. The newly prepared working culture and control tubes were placed in the shaking incubator for overnight observation, while the main tube was stored in the fridge at 4 °C.

#### Glycerol stock culture preparation

To preserve the bacterial strain for long-term use, glycerol stocks were prepared using a 1:3 ratio of glycerol to bacterial suspension. First, 5 mL of autoclaved glycerol was transferred into a sterile tube, followed by 15 mL of bacterial suspension. The mixture was gently swirled until homogeneous. The total 20 mL suspension was then aliquoted into 20 small sterile stock culture tubes. Each tube was sealed with parafilm and stored in a designated rack inside a − 20 °C freezer for long-term preservation.

#### Cleaning and laboratory maintenance

After completing the working culture preparation and stock preservation, the workspace was thoroughly cleaned. All used equipment was disinfected, and the lab was maintained under aseptic conditions for future experiments.

### Sample preparation for biofilm investigation

#### Initial bacterial growth observation

The newly prepared working cultures were observed the following day to assess growth. The control tube remained clear, while the experimental tube appeared slightly cloudy. However, the cloudiness was insufficient, and the absorbance reading was belowe the required range of 0.3–0.8. To ensure adequate bacterial growth, the experimental culture was returned to the shaking incubator for further incubation until a visibly denser bacterial suspension was obtained and the absorbance reading reached the desired range. Subsequent observations confirmed that the experimental tube became consistently cloudy over several days, indicating stable bacterial proliferation.

#### Bacterial growth quantification

To determine the absorbance reading, three cuvettes were prepared: one containing distilled water as a reference, another with blank media as a control, and the last with the bacterial suspension sample (Fig. 1g). Using a 1 mL pipette, 2 mL of each solution was carefully transferred into its respective cuvettes. The cuvettes were transported to the spectrophotometer (UV-1900i, Shimadzu), ensuring aseptic handling throughout, with hands disinfected using alcohol before and after handling the materials.

Upon arrival at the spectrophotometer, all three cuvettes’ outer clear surfaces were wiped clean to avoid any marks, residues, or scratches on the surfaces that could interfere with the light passage and result in inaccurate absorbance readings. Once cleaned, the instrument cover was opened, and the distilled water cuvette was placed at the reference position at the end of the holder. The blank media cuvette was positioned in front of it, and the spectrophotometer was set to a wavelength of 600 nm. The Auto-Zero function was activated to calibrate the machine before measurement. Once the zeroing process was completed, the blank media cuvette was removed and replaced with the bacterial suspension sample cuvette. The measurement was initiated by pressing the Start function and recording the absorbance reading.

The bacterial suspension in the centrifuge tube exhibited an absorbance reading of approximately 0.3, which was deemed suitable for further experimentation. After measurement, the cuvette was carefully removed by holding it at the top in a vertical position to prevent contamination. All used cuvettes were placed in a designated container and fully immersed in a disinfectant solution overnight to ensure proper decontamination.

#### Biofilm formation setup

Following bacterial quantification, the bacterial suspension from the centrifuge tube was aseptically transferred into a sterile petri dish (Fig. 1h). The as-received 5052 aluminium substrate was cut into small coupons with dimensions of 25 mm in length × 25 mm in width × 1 mm in thickness using a guillotine machine, sterilized with an autoclave machine, and then placed inside the petri dish to serve as a surface for biofilm attachment. The oxide layer and surface roughness of aluminium can influence biofilm adhesion and growth^12^, making it an ideal candidate for studying biofouling behaviour in freshwater. The samples were incubated under static conditions in a static incubator (Shel Lab), simulating biofilm formation in natural aquatic environments such as lakes and ponds. Proper labelling was conducted for identification, and biofilm development was monitored over a period of two months.

### Biofilm removal from aluminium substrates using fiber laser machine

#### Biofilm development and pre-removal observation

Biofilm formation on aluminium substrates was observed after a two-month incubation period^13–15^. The biofilm development was visually monitored weekly. A thin white layer was appeared on the surface of the aluminium substrate, confirming the presence of biofilm.

#### Fiber laser removal process

**Fig. 3:**
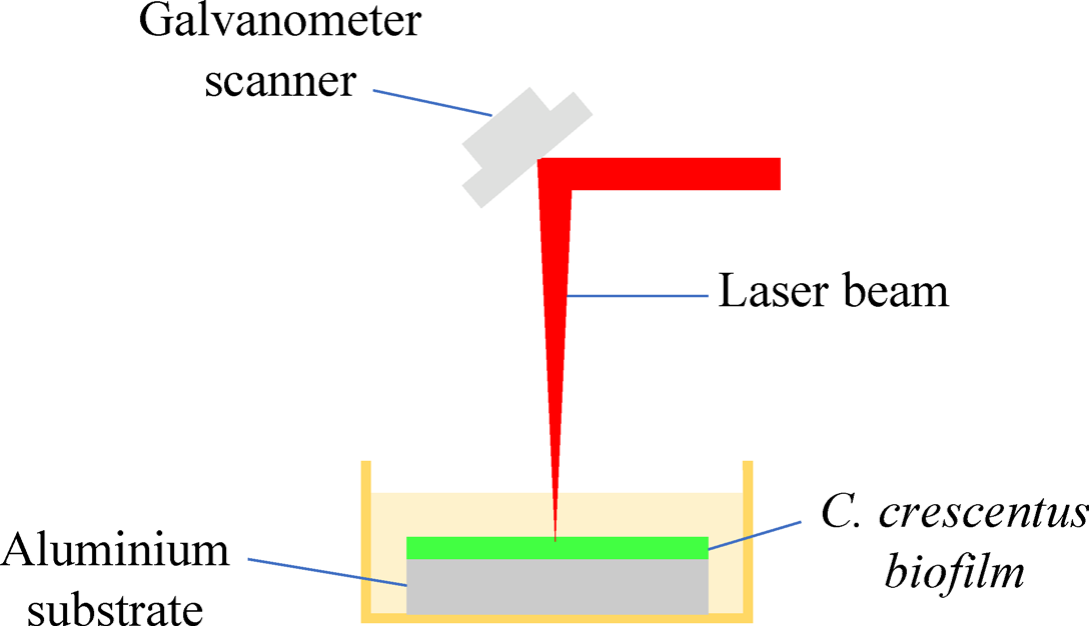
Experimental setup for underwater removal of biofilm.

The biofilm removal process was conducted using a 50 W fiber laser machine (CUM50F, Cloudray) operating at a 1064 nm wavelength (Fig. 3). The system utilizes a Q-pulsed laser with a pulse duration estimated of 120 ns and a Gaussian beam profile. The smallest focused spot diameter was approximately 65 μm when using the 290 mm focal length lens. A fixed pulse frequency of 20 kHz was maintained throughout all experiments. The standoff distance was set at 360 mm, with the sample positioned 3 mm below the water surface. Due to this configuration and beam divergence in water, the effective spot diameter at the sample surface was estimated to be approximately 600 μm. The laser power was varied at 70%, 80%, and 90% of the maximum 50 W output, corresponding to estimated fluence values of 619.1 mJ/cm^2^, 707.6 mJ/cm^2^, and 796.1 mJ/cm^2^, respectively. These fluence values were derived from the pulse energy, pulse frequency, and beam area to enhance reproducibility and accurately reflect the energy applied during treatment.

In the preliminary experiments, it was observed that when the number of repetition loops reached seven or exceeded five, excessive material removal occurred, causing damage to the substrate surface. Conversely, at laser power levels below 70%, no visible marking or biofilm removal was observed. Additionally, when the laser speed was too high, the interaction time was insufficient, resulting in ineffective biofilm removal. Based on the preliminary results, laser power was set at 70%, 80%, and 90% of the 50 W capacity, while laser speed was adjusted to 5 mm/s, 10 mm/s, and 15 mm/s. Additionally, the number of laser repetition loops was varied between 1, 3, and 5 to evaluate the effectiveness of multiple repetitions. It is noted that laser scanning was performed in a unidirectional manner, where each repetition loop began at the same starting point without bidirectional movement, ensuring consistent energy input and avoiding overlap between passes. Each combination of these parameters was tested systematically using a second-order Box-Behnken Design (BBD) to evaluate its efficiency in removing the biofilm layer from the aluminium substrate. It is noted that the standoff distance was set at 360 mm, and the sample was submerged 3 mm below the liquid surface.

### Post-removal analysis method

#### Visual inspection of surface changes

**Fig. 4:**
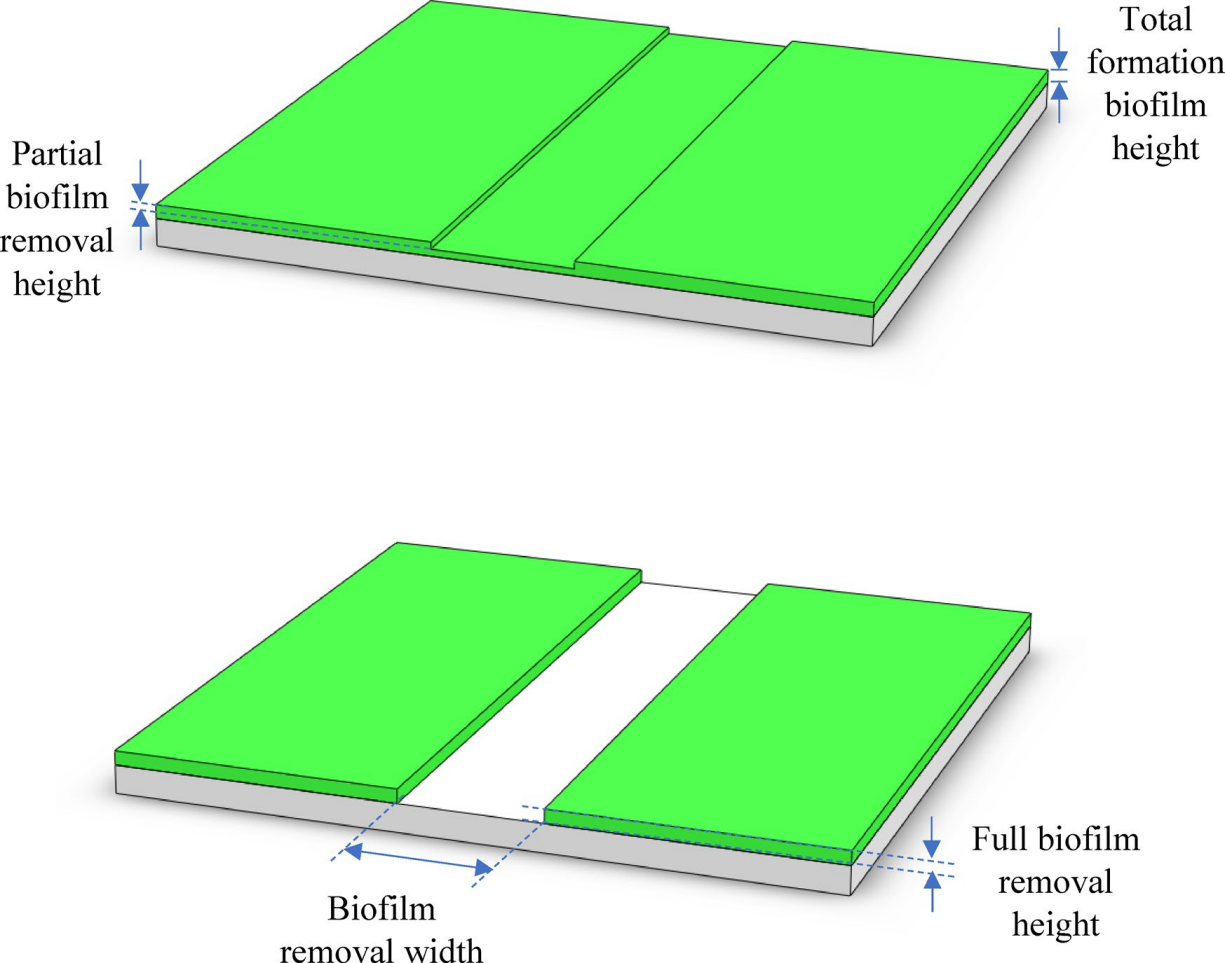
Microscope image of laser-treated aluminium surface showing green-stained residual biofilm. The areas without green staining indicate regions where biofilm was successfully removed, used to assess biofilm removal width.

Following laser treatment, the aluminium substrates were visually examined to assess the effectiveness of biofilm removal and any noticeable surface alterations. The evaluation focused on identifying residual biofilm, discoloration, and oxidation effects caused by laser exposure. Discoloration appeared in the form of light brown, dark brown, or bluish hues, depending on the laser intensity. Oxidation effects, such as the formation of a white or grayish powdery layer, were also observed. To improve visualization and measurement accuracy, the residual biofilm was dyed green after laser treatment (Fig. 4), making the biofilm removal width more distinguishable under a digital microscope (Hirox RH-2000).

#### Quantification of biofilm removal (total removal height measurement)

**Fig. 5:**
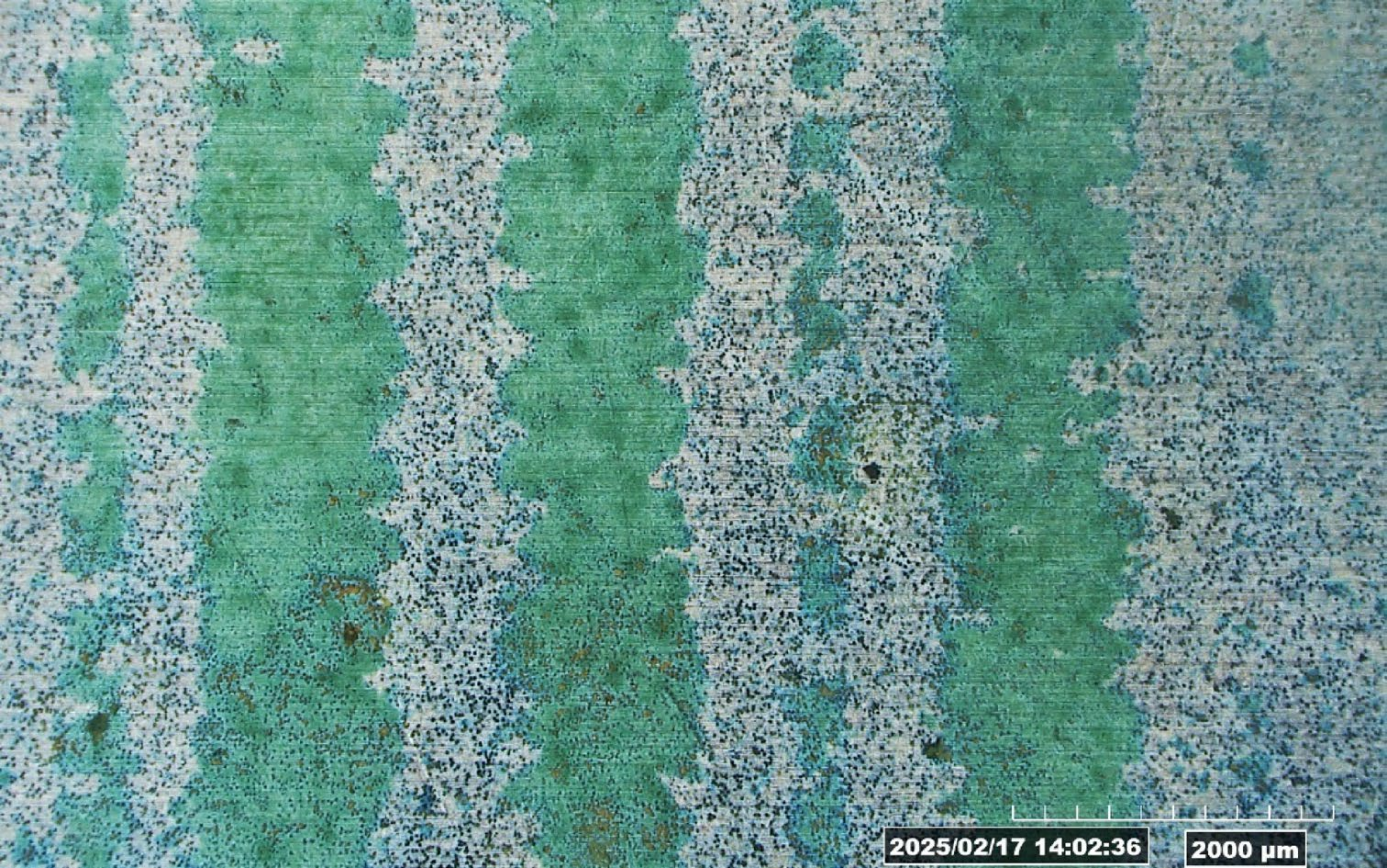
Biofilm removal height and width diagrams.

To quantify biofilm removal effectiveness, the total removal height was measured using the Hirox RH-2000 digital microscope. This measurement helped determine the extent of biofilm removal from the aluminium substrate (Fig. 5). Using the collected laser parameter settings and total removal height data, a BBD analysis was conducted in MATLAB to evaluate the relationships between laser parameters and biofilm removal effectiveness. This design included 15 experimental runs, with three replicates at the center point (80% laser power, 10 mm/s laser speed, 3 repetition loops) to estimate pure error.

## Results and discussion

In underwater laser treatment, several physical mechanisms likely help remove biofilm. First, thermal stress arises from rapid surface heating, causing expansion that loosens biofilm attachment^16^. Next, cavitation occurs when vapor bubbles form and collapse in the water, producing forces that help detach biofilm. Finally, recoil pressure from sudden vapor expansion or surface vaporization exerts a mechanical push-back against the biofilm. Although these interactions were not directly recorded due to limitations in instrumentation, the observed removal patterns are consistent with mechanisms reported in previous studies on submerged laser ablation^17^.

Beyond the physical forces involved, the surrounding environment also plays a critical role in shaping the biofilm’s thermal and mechanical response. In dry environments, heat loss is mainly driven by natural convection and the initial surface temperature of the biofouling layer, often resulting in uneven thermal loading and the development of internal stresses^18,19^. These residual stresses can compromise the mechanical integrity of both the substrate and the biofilm. By contrast, underwater laser processing benefits from the higher thermal conductivity and heat capacity of water, which helps to dissipate heat more uniformly and reduce thermal stress. When the biofilm is modelled as a cylindrical elastic rod, temperature-dependent material behaviour and isotropic elasticity suggest that laser exposure in a submerged setting can limit excessive deformation and mechanical fatigue^20^.

In this study, a second-order BBD with three factors and three levels was employed, resulting in a total of 15 experimental runs, including three repeated center points (80% of 50 W laser power, 10 mm/s laser speed, 3 repetition loops) to enhance statistical reliability. The selected parameters and their respective values used in the experimental design are detailed in Table 1. This design allows for the evaluation of both linear and interaction effects of the laser parameters on biofilm removal efficiency while maintaining a balanced distribution of experimental conditions across the defined factor space.

**Table 1:** The value setting for the laser’s parameters (3 factors, 3 levels).

	Minimum	Median	Maximum
Laser power (% of 50 W)	70	80	90
Laser speed (mm/s)	5	10	15
Repetition loop (unitless)	1	3	5

Based on the data provided in Table 2, three experiments exhibited a white line along the laser-marked region, visible both to the naked eye and under a microscope. While its exact nature remains inconclusive, it may be attributed to oxidation effects, material removal, or a combination of both due to the laser’s interaction with the aluminium substrate. Further investigation, such as depth measurement comparisons, surface roughness analysis, or cross-sectional imaging, would be necessary to definitively distinguish between oxidation and material removal mechanisms in fiber laser biofilm removal on aluminium substrates.

**Table 2:** The Box-Behnken experimental data.

Experiment	Laser power (%)	Laser speed (mm/s)	Repetition loop	Markable	Abnormality
1	80	10	3	–	X
2	80	5	1	–	X
3	80	5	5	–	White line
4	80	15	1	–	X
5	80	15	5	–	X
6	70	10	1	–	X
7	80	10	3	–	X
8	70	10	5	–	X
9	90	10	1	–	X
10	80	10	3	–	X
11	90	10	5	–	White line
12	70	5	3	–	X
13	70	15	3	–	X
14	90	5	3	–	White line
15	90	15	3	–	X

Table 3 and its Supplementary Table 1 present the biofilm removal depth data obtained from fiber laser treatment under varying experimental conditions. The removal depth was measured to evaluate the efficacy of different laser parameter settings in eliminating biofilm from the aluminium substrate. The results indicate that removal depth varied significantly depending on laser power, laser speed, and the number of repetition loops, with deeper removal observed at higher laser power settings or increased repetition loops. In contrast, lower laser power and higher laser speed settings resulted in minimal biofilm removal, reinforcing the importance of parameter optimization.

The removal depth percentage was calculated based on the initial biofilm thickness, allowing for a comparative analysis of biofilm removal efficiency across different settings. The highest removal percentage was observed at high laser power (90%), medium laser speed (10 mm/s), and one repetition loop, where biofilm removal exceeded 94.71%. Conversely, the lowest removal was recorded at low laser power (70%) and medium laser speed (10 mm/s), where the biofilm removal percentage remained below 38.36%. These findings highlight the significance of optimizing laser parameters to achieve effective biofilm removal while minimizing potential damage to the aluminium substrate.

**Table 3:** The biofilm removal depth data.

No.	Experiment sequence	Laser power (%)	Laser speed (mm/s)	Laser repetition loop	Average removal depth (± 0.001 mm)	Removal depth (%)
1	12	70	5	3	0.267	64.91
2	13	70	15	3	0.317	77.22
3	14	90	5	3	0.367	89.54
4	15	90	15	3	0.258	63.06
5	6	70	10	1	0.183	38.36
6	8	70	10	5	0.442	93.12
7	9	90	10	1	0.450	94.71
8	11	90	10	5	0.125	30.42
9	2	80	5	1	0.317	72.97
10	3	80	5	5	0.333	76.68
11	4	80	15	1	0.392	90.36
12	5	80	15	5	0.308	71.34
13	1	80	10	3	0.117	26.91
14	7	80	10	3	0.358	75.40
15	10	80	10	3	0.383	80.69

### Regression model and response surface plot analysis for biofilm removal depth

The BBD was used to evaluate the effects of laser power, laser speed, and repetition loop on biofilm removal depth in an underwater laser removal process. The regression model included linear, interaction, and quadratic terms, however, with an R² of 0.718 and a non-significant p-value (0.366), its overall predictive capability was limited.

Among the tested factors (Table 4), repetition loop was the only statistically significant variable (*p* = 0.0407), demonstrating a strong influence on biofilm removal. Additionally, its interaction with laser power (*p* = 0.0279) was significant, indicating that higher laser power levels combined with multiple repetition loops improve biofilm removal efficiency. Conversely, laser power and laser speed were not statistically significant (*p* = 0.3645), suggesting that their individual and combined effects were weak or masked by other dominant factors.

**Table 4:** Estimated coefficients data for biofilm removal depth.

Parameter	Estimate	Standard error	t-statistic	*p*-value
Intercept	− 438.600	677.890	− 0.65	0.5462
Laser power	7.269	16.356	0.44	0.6753
Laser speed	6.588	17.796	0.37	0.7264
Repetition loop	117.640	42.920	2.74	0.0407
Laser power × Laser speed	− 0.194	0.194	− 1.00	0.3645
Laser power × Repetition loop	− 1.488	0.485	− 3.07	0.0279
Laser speed × Repetition loop	− 0.570	0.970	− 0.59	0.5825
Laser power squared	− 0.005	0.101	− 0.05	0.9615
Laser speed squared	0.528	0.404	1.31	0.2485
Repetition loop squared	0.916	2.525	0.36	0.7317

**Fig. 6:**
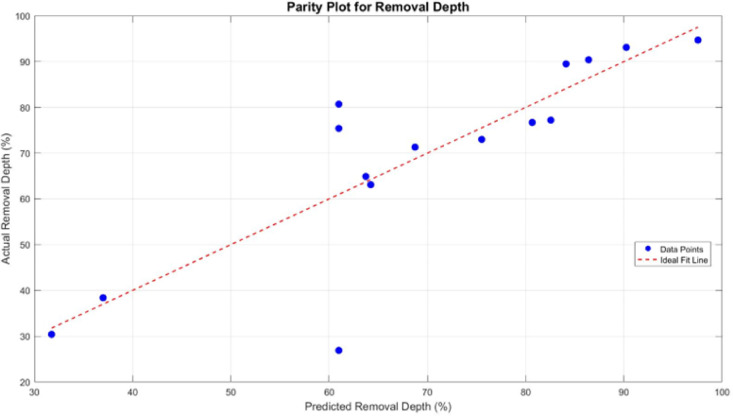
The parity plot for removal depth.

**Fig. 7:**
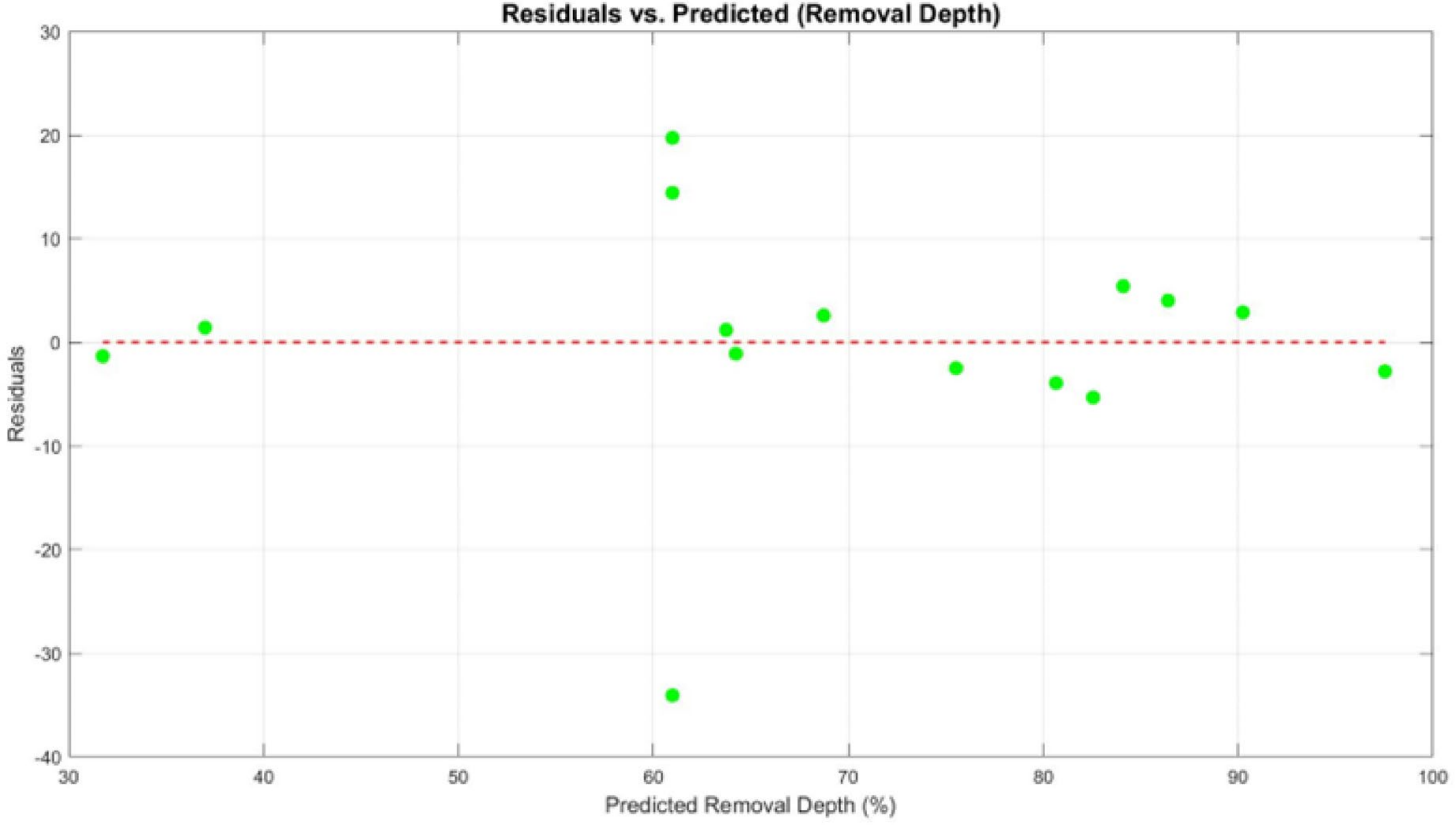
The residuals vs. predicted removal depth.

**Fig. 8:**
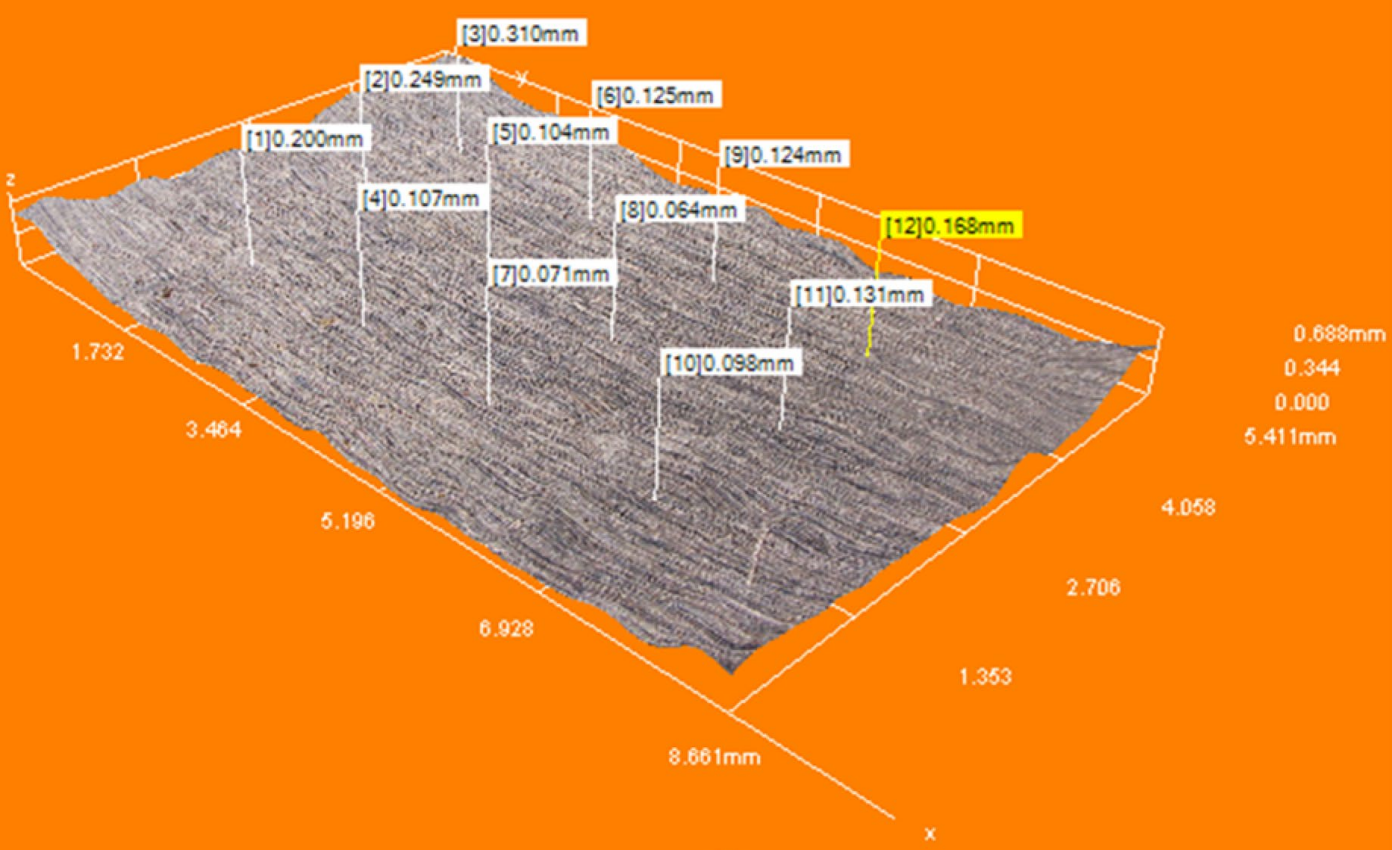
3D height profile of the bare aluminium substrate.

To evaluate the model’s accuracy and reliability, a parity plot and residual analysis were carried out for the biofilm removal depth. In the parity plot (Fig. 6), most data points fall close to the ideal reference line, indicating reasonable agreement between the predicted and experimental values. However, a few points show noticeable deviation, which suggests some level of model inaccuracy under certain parameter combinations. The residual plot (Fig. 7) provides additional insight. While the residuals are generally scattered around zero, there is no clear pattern, which implies that the model does not suffer from systematic error. That said, the spread of residuals, especially the presence of both high positive and negative values, highlights areas where the model prediction is less stable. This is consistent with the relatively low adjusted R^2^ value reported, suggesting that although the model captures the trend, it may not account for all variations in the response. This actually can be explained by variations in height as shown in Fig. 8, indicating inconsistencies in the surface profile of the bare aluminium substrate. These differences in surface roughness affected the formation of the biofilm, leading to variations in biofilm height and, consequently, uneven biofilm removal depth. The inconsistencies can be attributed to manufacturing process variability, where differences in the substrate’s thickness across the sample contribute to non-uniform surface characteristics. As a result, the laser interacts differently with various biofilm regions, impacting the overall removal efficiency and depth uniformity.

Confidence intervals (CI) for each model term (Table 5) for biofilm removal depth were calculated at the 95% level to evaluate the reliability of coefficient estimates. Terms with intervals that do not cross zero, such as interaction between laser power and repetition loop, suggest statistically significant influence within the tested range. In contrast, wide intervals or those that span zero, as seen in most other terms, indicate limited predictive certainty. This suggests that while the model captures overall trends, its term-level contributions should be interpreted with caution.

**Table 5:** ANOVA confidence intervals data for biofilm removal depth.

Parameter	Estimate	Lower 95% CI	Upper 95% CI
Intercept	− 438.597	− 2181.178	1303.984
Laser power	7.269	− 34.775	49.312
Laser speed	6.588	− 39.160	52.335
Repetition loop	117.644	7.315	227.973
Laser power × Laser speed	− 0.194	− 0.692	0.305
Laser power × Repetition loop	− 1.488	− 2.735	− 0.240
Laser speed × Repetition loop	− 0.570	− 3.065	1.925
Laser power squared	− 0.005	− 0.265	0.255
Laser speed squared	0.528	− 0.511	1.566
Repetition loop squared	0.916	− 5.575	7.406

**Fig. 9:**
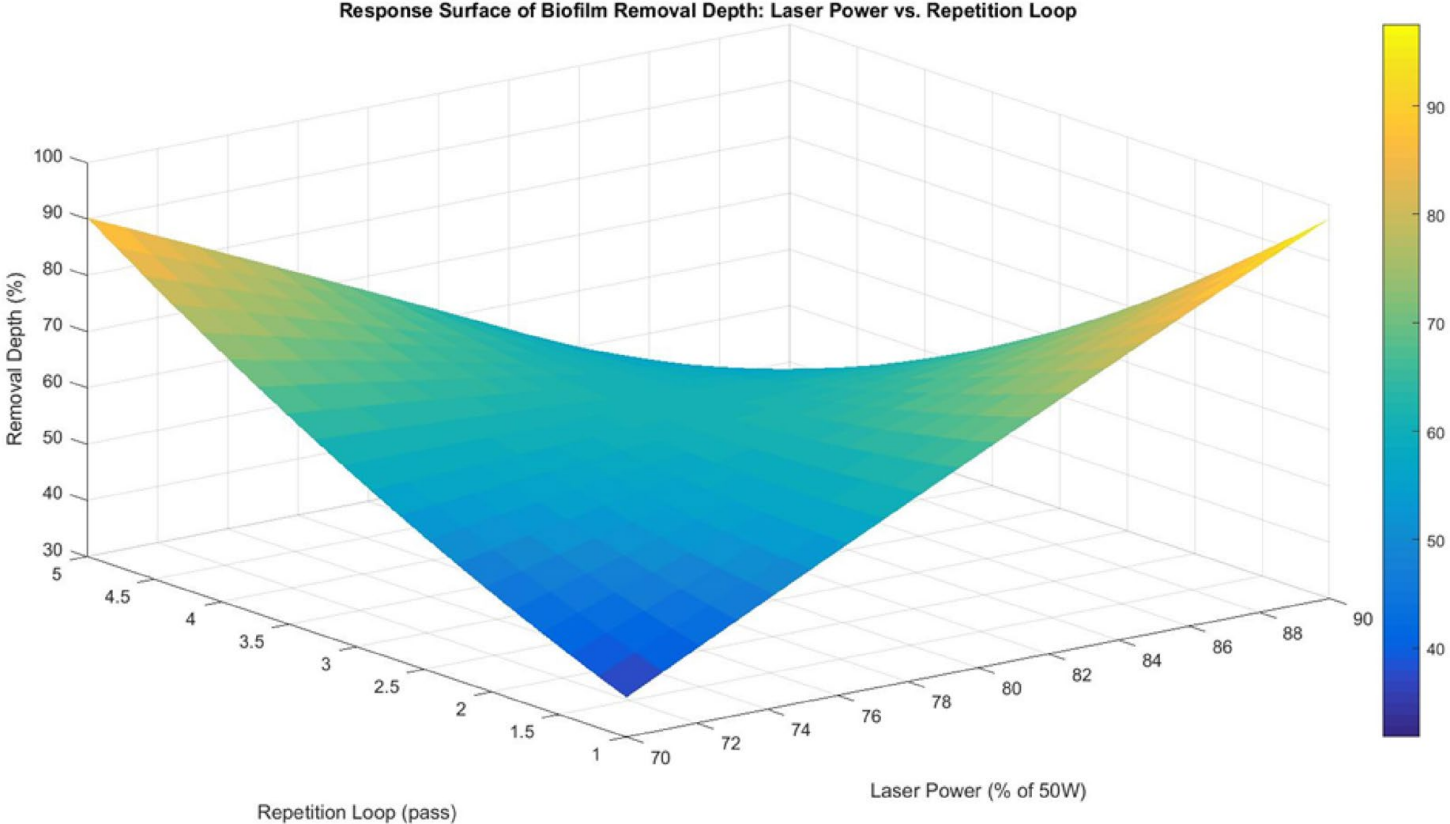
Response surface for biofilm removal depth between laser power and repetition loop.

The response surface between laser power and repetition loop (Fig. 9) confirms the regression findings, showing a significant increase in biofilm removal depth with higher repetition loops, particularly at higher laser power levels. At low repetition loops, biofilm removal is minimal, as a single repetition loop is insufficient to overcome biofilm adhesion and hydration barriers. As repetition loops increase, progressive weakening of biofilm adhesion facilitates detachment, with repeated heating cycles reducing biofilm integrity and enhancing removal efficiency. The interaction suggests that higher laser power enhances the effect of multiple repetition loops, as it provides more localized heat and compensates for energy losses due to water absorption and heat dissipation by aluminium. This trend reinforces the hypothesis that underwater laser processing requires repeated exposure to mitigate thermal diffusion losses^21,22^, especially when applied to biofilms on high-conductivity substrates like aluminium.

**Fig. 10:**
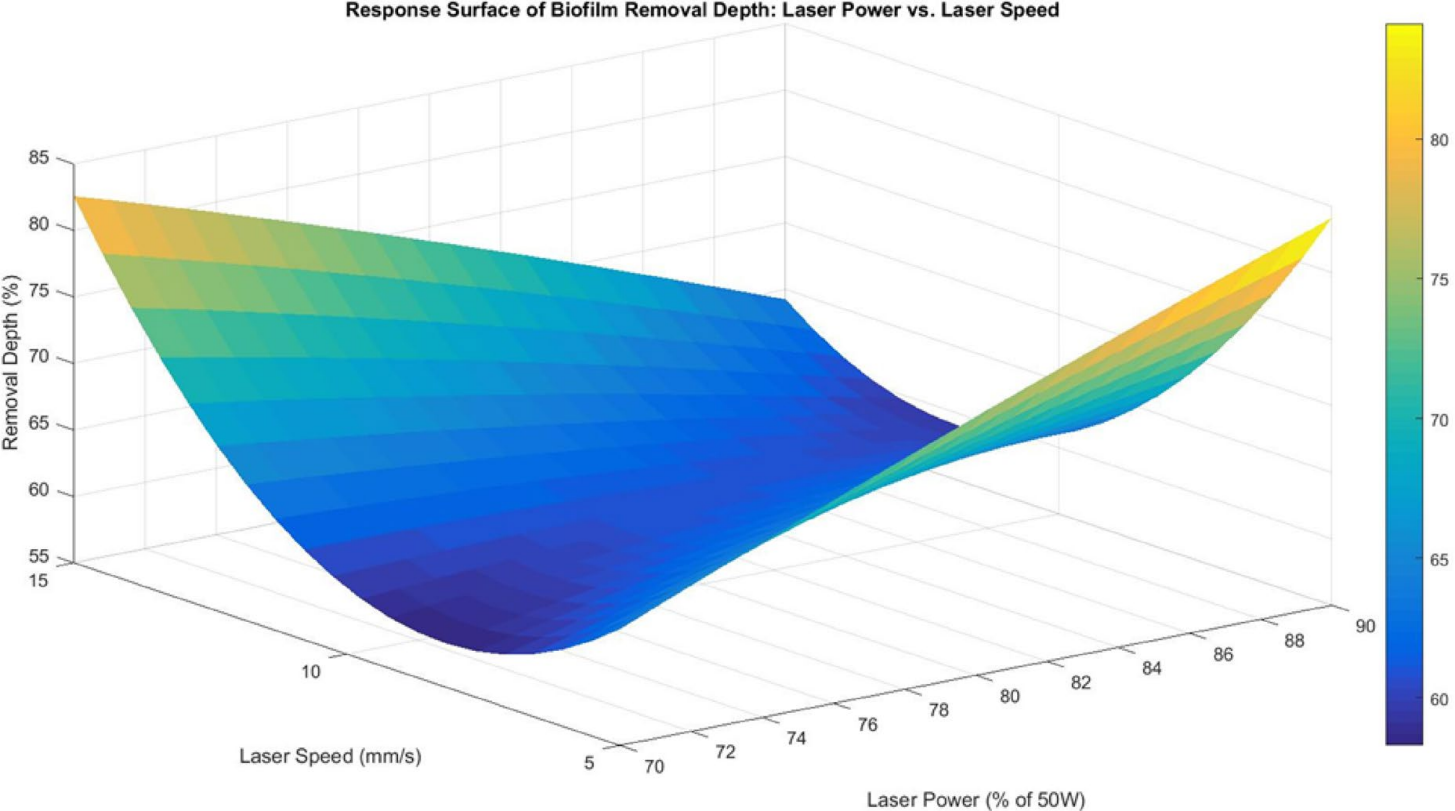
Response surface for biofilm removal depth between laser power and laser speed.

The response surface between laser power and laser speed (Fig. 10) exhibits a U-shaped curvature, where removal depth is lowest at the mid-range laser power of 80% and lower laser speed of 5 mm/s, but increases at both higher and lower laser power levels. However, neither factor was statistically significant, suggesting that the observed variations were likely influenced by external interactions rather than direct factor relationships. One possible explanation is energy attenuation and scattering in the submerged system. It is noted that fiber lasers operating at a 1064 nm wavelength exhibit low absorption in water, allowing some laser energy to penetrate the water layer before reaching the biofilm, leading to attenuation and scattering, which reduce its direct impact. Another contributing factor is microbubble formation caused by localized vaporization, which scatters or blocks the laser beam, resulting in inconsistent energy delivery. This effect is particularly noticeable in center-point replications, where small fluctuations in bubble formation, biofilm adhesion, or surface roughness may have affected consistency. Additionally, aluminium’s high thermal conductivity prevents heat accumulation in the laser impact zone, which is essential for uniform biofilm detachment. As heat spreads rapidly through the substrate, the expected impact of higher laser power or slower laser speed is diminished. The U-shaped response trend suggests that extreme settings (either high or low laser power) are more effective than moderate conditions, likely due to fluctuations in biofilm response to laser heating. However, the lack of statistical significance indicates that these variations may be influenced by uncontrolled experimental conditions rather than a direct cause-effect relationship.

**Fig. 11:**
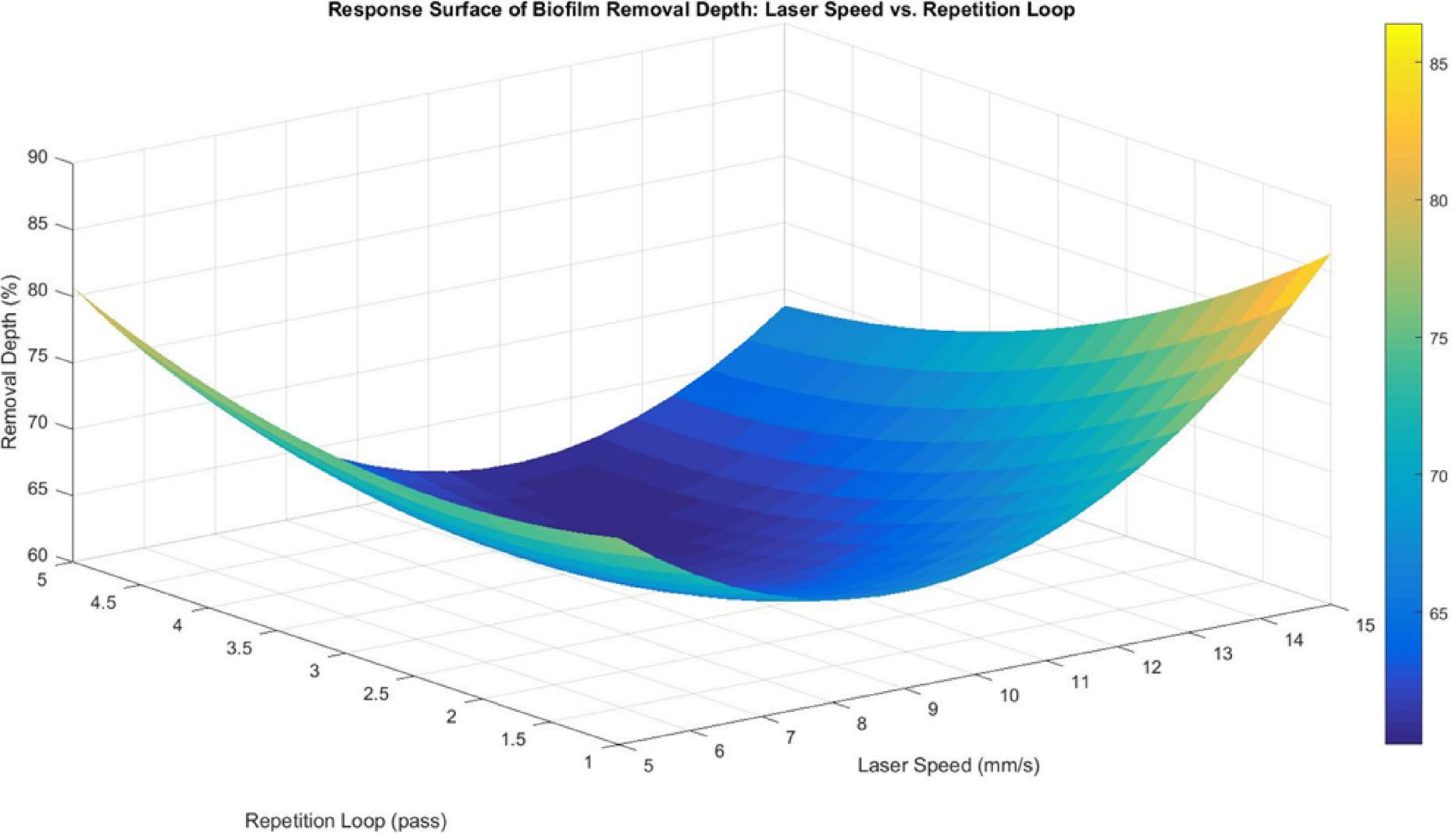
Response surface for biofilm removal depth between laser speed and repetition loop.

The response surface between laser speed and repetition loop (Fig. 11) further reinforces the dominant role of the repetition loop, while the effect of laser speed remains weak and inconsistent. At lower repetition loops, biofilm removal is minimal, confirming that a single laser repetition loop is insufficient to overcome biofilm hydration and adhesion strength. As repetition loops increase, removal depth improves significantly, supporting the idea that successive laser cycles gradually degrade biofilm adhesion and enhance detachment efficiency. However, laser speed does not show a clear correlation with removal efficiency, suggesting that water-induced scattering and aluminium heat dissipation override the expected trends in laser-material interactions. In a submerged system, increasing laser speed does not necessarily reduce energy deposition as it might in conventional laser applications, since beam scattering and heat dissipation counteract its effects. This supports the regression findings, where repetition loop was the only significant factor, and laser speed had no substantial impact on biofilm removal.

The results suggest that the repetition loop is the most critical factor for effective biofilm removal, as multiple repetition loops compensate for energy losses caused by water absorption and aluminium heat dissipation. Higher laser power enhances this effect by delivering more concentrated energy across multiple cycles, although its individual effect is limited. Laser speed, contrary to expectations, does not significantly influence biofilm removal, likely due to water-induced scattering and thermal dispersion that disrupt energy accumulation. These findings indicate that underwater laser processing requires repeated exposure to overcome the challenges posed by water and thermal diffusion losses.

The highest predicted biofilm removal depth of 114.95 μm was obtained at 90% laser power, 5 mm/s laser speed, and 1 repetition loop, based on the fitted response surface model (Table 6). This parameter set was not included in the original experimental runs and therefore represents a model-based estimation rather than a direct observation. Reproducing the exact biofilm structure for additional validation is not feasible, as factors such as bacterial count and growth rate can vary despite following the same culturing protocol (Fig. 1). These variations are often influenced by environmental and biological factors beyond experimental control. For this reason, no additional experimental runs were conducted to match the predicted parameter set. The same consideration applies to the predicted maximum value for biofilm removal width.

**Table 6:** Summary of the optimized laser parameters and predicted biofilm removal depth.

Parameter	Optimized value	Predicted depth (µm)	Note
Laser power (% of 50 W)	90	114.95	Based on fitted regression model
Laser speed (mm/s)	5		
Repetition loop	1		

Table 7 and its Supplementary Table 2 present the biofilm removal width data obtained from fiber laser treatment under varying parameter settings. The width of the removed biofilm region was measured to assess the lateral spread of laser interaction on the aluminium substrate, providing insights into how laser power, laser speed, and repetition loops influence the extent of biofilm removal beyond the targeted area.

The results indicate that higher laser power and lower laser speed generally lead to a wider removal width, with the maximum recorded width being 1.316 mm at 90% laser power, 5 mm/s laser speed, and 3 repetition loops. Conversely, lower laser power and higher laser speed resulted in a more confined removal area, with the minimum width of 0.423 mm observed at 70% laser power, 10 mm/s laser speed, and 1 repetition loop. Additionally, an increase in the number of laser repetition loops slightly expanded the removal width, as seen in the 0.820 mm width recorded at 5 repetition loops, compared to 0.574 mm at a single repetition loop for the same laser power (80%) and same laser speed (5 mm/s), suggesting cumulative heat effects and prolonged laser-material interaction. Understanding these variations is essential for optimizing laser parameters to achieve precise biofilm removal while minimizing unintended exposure and potential substrate damage^23^.

**Table 7:** The biofilm removal width data.

No.	Experiment sequence	Laser power (%)	Laser speed (mm/s)	Laser repetition loop	Average removal width (± 0.001 mm)
1	12	70	5	3	0.824
2	13	70	15	3	0.497
3	14	90	5	3	1.316
4	15	90	15	3	0.798
5	6	70	10	1	0.423
6	8	70	10	5	0.634
7	9	90	10	1	0.598
8	11	90	10	5	0.931
9	2	80	5	1	0.574
10	3	80	5	5	0.820
11	4	80	15	1	0.495
12	5	80	15	5	0.594
13	1	80	10	3	0.692
14	7	80	10	3	0.620
15	10	80	10	3	0.584

Similarly to biofilm removal depth, the BBD was used to evaluate the effects of laser power, laser speed, and repetition loop on biofilm removal width in an underwater fiber laser removal process (Table 8). The fitted regression model included linear, interaction, and quadratic terms, achieving an R^2^ value of 0.913, indicating that the model explains a significant portion of the variability in removal width. The model’s F-statistic (5.83, *p* = 0.0333) confirms that the overall model is statistically significant.

**Table 8:** Estimated coefficients for biofilm removal width.

Parameter	Estimate	Standard error	t-statistic	*p*-value
Intercept	7.210	3.837	1.88	0.1190
Laser power	− 0.181	0.093	− 1.96	0.1076
Laser speed	− 0.022	0.101	− 0.22	0.8378
Repetition loop	0.138	0.243	0.57	0.5947
Laser power × Laser speed	− 0.001	0.001	− 0.87	0.4244
Laser power × Repetition loop	0.002	0.003	0.55	0.6026
Laser speed × Repetition loop	− 0.004	0.005	− 0.67	0.5331
Laser power squared	0.001	0.001	2.21	0.0782
Laser speed squared	0.004	0.002	1.76	0.1391
Repetition loop squared	− 0.028	0.014	− 1.95	0.1080

However, despite the model’s significance, individual process parameters did not exhibit strong direct effects on removal width. Instead, the quadratic terms of laser power and repetition loop indicate a non-linear relationship, suggesting that removal width expands up to a certain point before stabilizing. Notably, laser power approached significance (*p* = 0.1076), indicating a moderate influence on width expansion, while its quadratic term (*p* = 0.0782) suggests that higher laser power levels contribute to width expansion but with diminishing returns. Similarly, the quadratic effect of repetition loop (*p* = 0.108) implies that additional laser repetition loops increase width initially but reach a plateau as previously removed regions prevent further lateral growth.

**Fig. 12:**
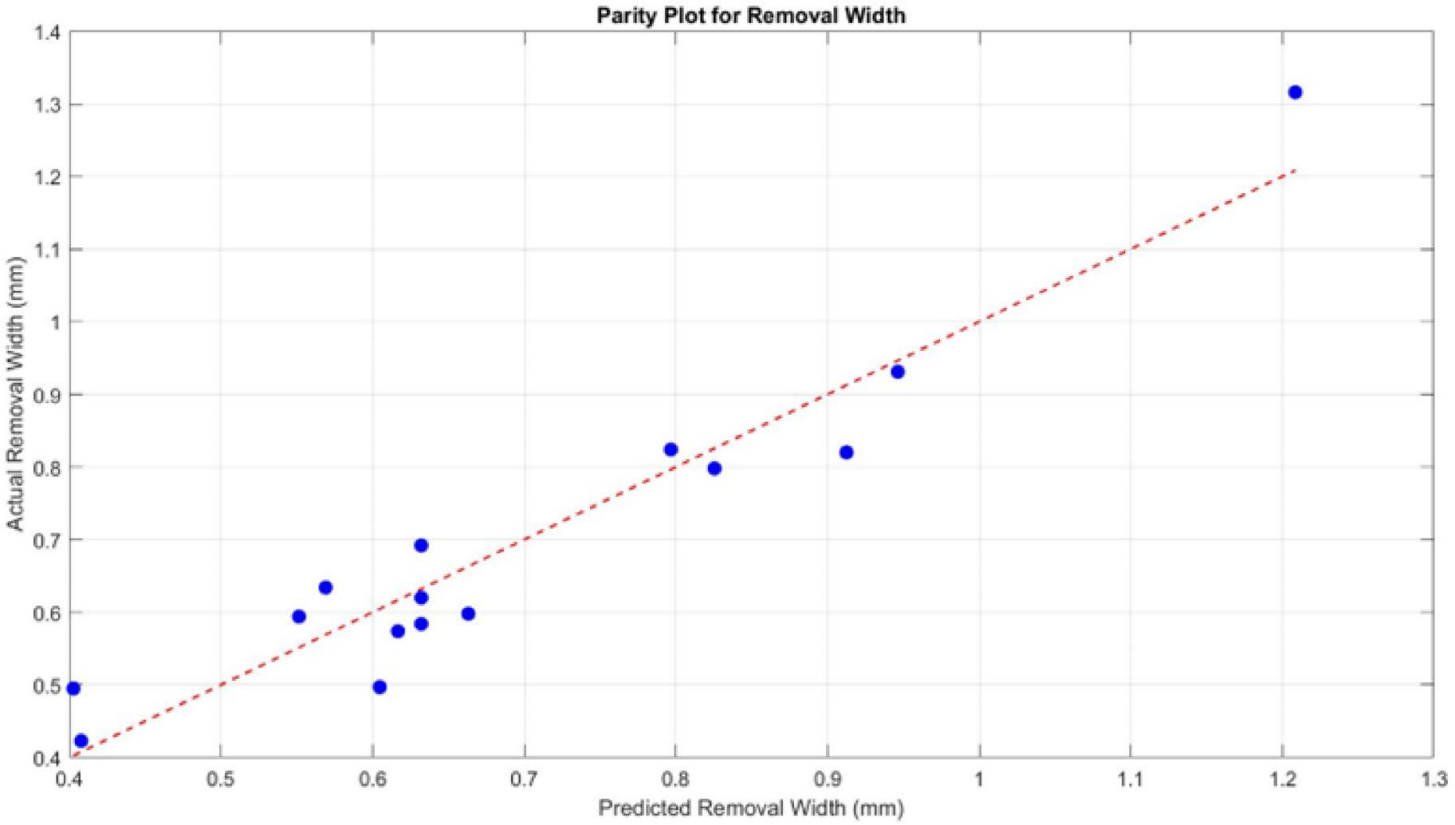
The parity plot for removal width.

**Fig. 13:**
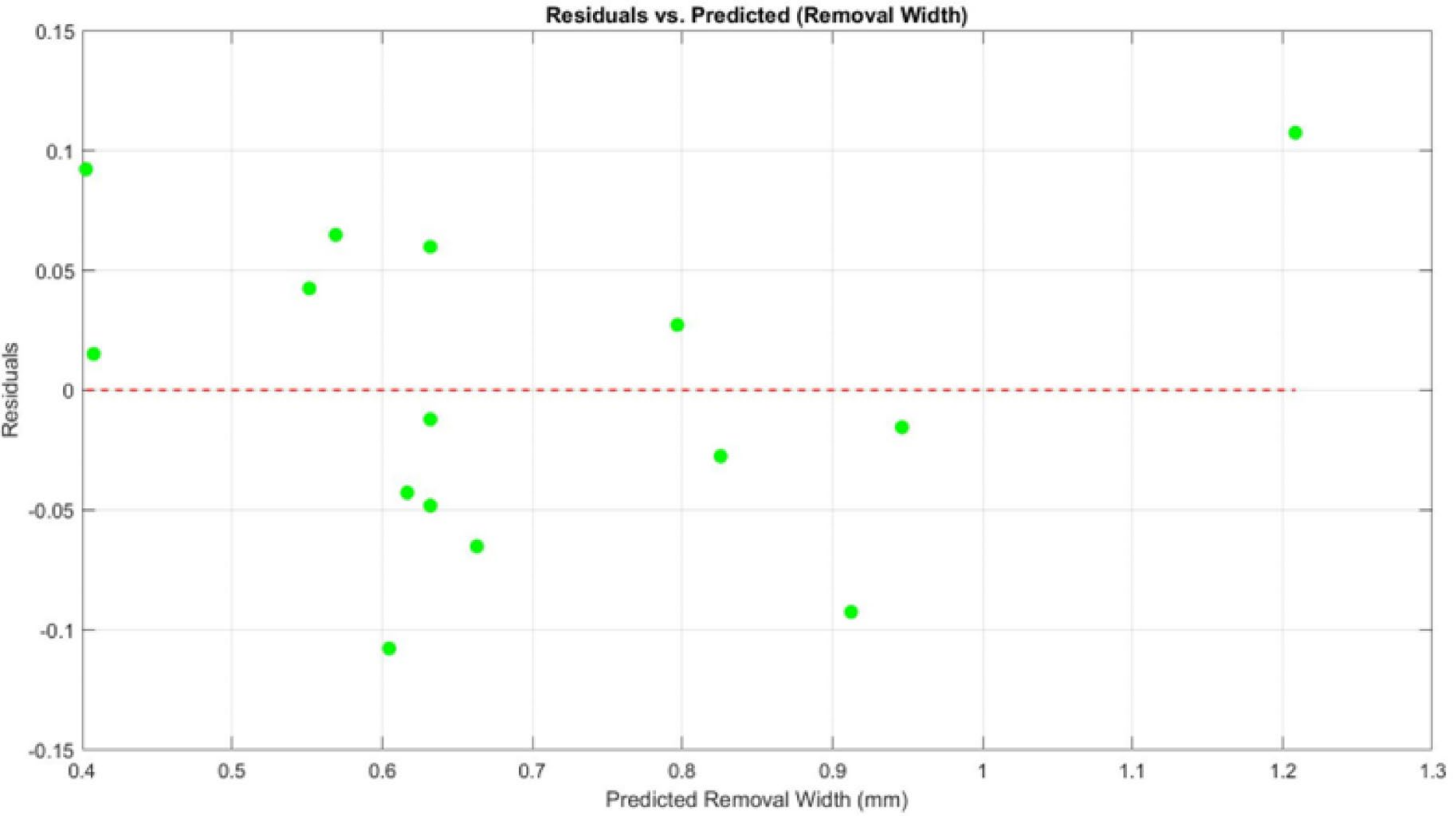
The residuals vs. predicted removal width.

To verify the model’s performance for biofilm removal width, a parity plot and residual analysis were performed. In the parity plot (Fig. 12), the predicted values align closely with the actual measurements, with minimal deviation from the reference line. This indicates a strong match between model outputs and experimental results. The residual plot (Fig. 13) further supports the model’s reliability. Residuals are small and randomly distributed around zero, with no apparent trend or clustering. This suggests the model captures the response behaviour well across the tested parameter space. The high R^2^ (0.913) and adjusted R^2^ (0.756) values further confirm the model’s predictive strength and consistency.

The 95% CI (Table 9) provides insight into the reliability of each model term for biofilm removal width. Several terms, particularly the intercept and quadratic components, have narrow intervals that do not cross zero, indicating stable contributions to the model. Although a few linear and interaction terms have wider intervals that include zero, the high R^2^ (0.913) and adjusted R^2^ (0.6954) suggest the model effectively captures the response behaviour across the tested range.

**Table 9:** ANOVA confidence intervals data for biofilm removal width.

Parameter	Estimate	Lower 95% CI	Upper 95% CI
Intercept	7.210	− 2.653	17.073
Laser power	− 0.181	− 0.419	0.057
Laser speed	− 0.022	− 0.281	0.237
Repetition loop	0.138	− 0.487	0.762
Laser power × Laser speed	− 0.001	− 0.004	0.002
Laser power × Repetition loop	0.002	− 0.006	0.009
Laser speed × Repetition loop	− 0.004	− 0.018	0.010
Laser power squared	0.001	− 0.000	0.003
Laser speed squared	0.004	− 0.002	0.010
Repetition loop squared	− 0.028	− 0.065	0.009

**Fig. 14:**
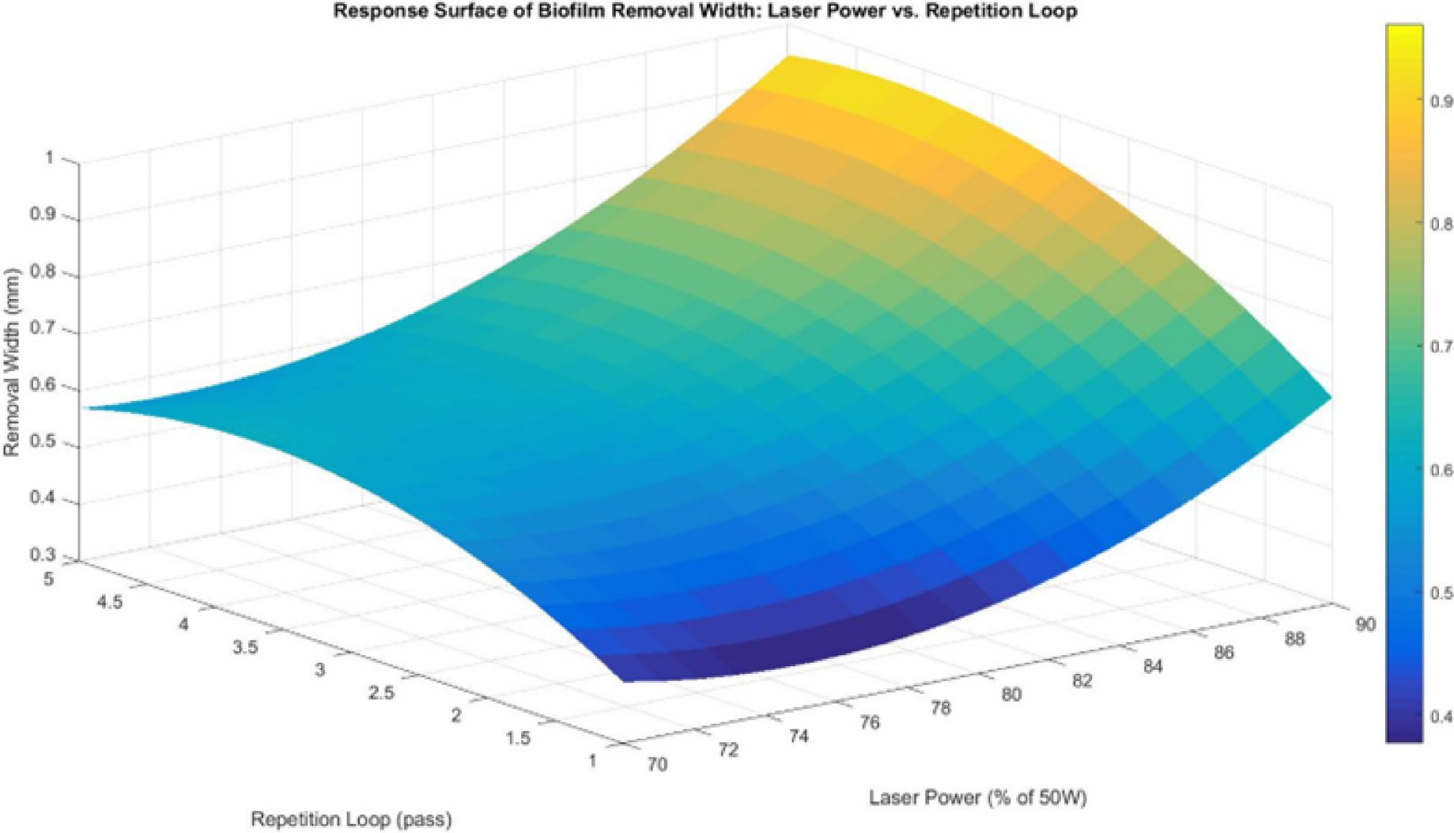
Response surface for biofilm removal width between laser power and repetition loop.

The response surface between laser power and repetition loop (Fig. 14) confirms this non-linear trend, showing that higher laser power and repetition loop values increase removal width, but the effect diminishes at higher levels. The significance of the laser power quadratic term supports this, indicating that thermal diffusion within the biofilm expands the affected area, but water and aluminium as heat sinks limit excessive width growth. Since water acts as an energy dissipator, excess energy beyond a certain point does not effectively contribute to lateral expansion. Furthermore, the repetition loop does not have a strong impact on width, as suggested by the regression model. This may be because repetition loops primarily contribute to deeper ablation rather than lateral expansion, meaning that additional loops do not significantly alter width once an area has been removed^24^.

**Fig. 15:**
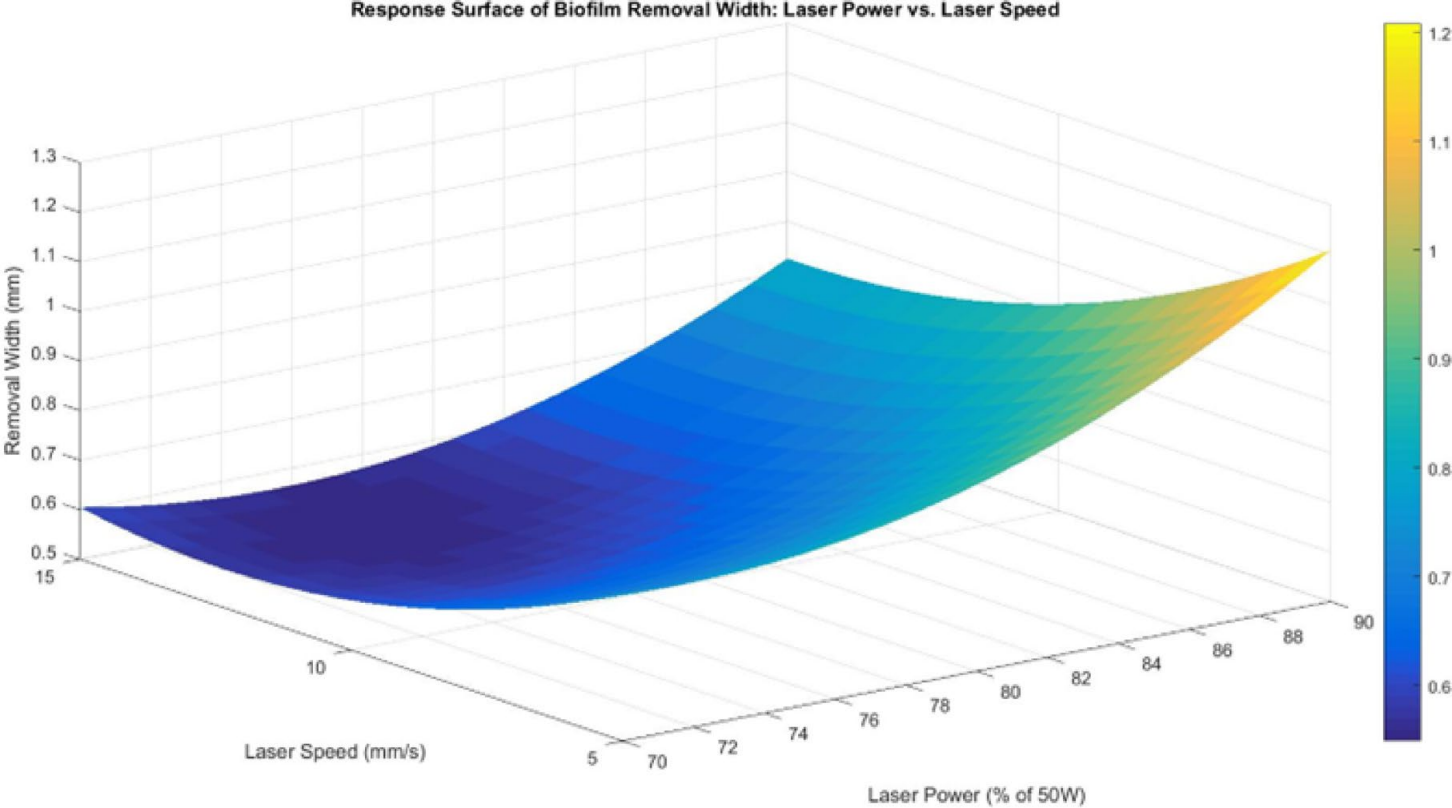
Response surface for biofilm removal width between laser power and laser speed.

The response surface between laser power and laser speed (Fig. 15) exhibits a curved trend, where removal width increases with higher laser power but varies inconsistently with laser speed. This aligns with the quadratic nature of laser power, reinforcing that width expansion is not linearly proportional to power but follows a more complex trend. One explanation for this effect lies in the laser’s interaction with the water layer. Since 1064 nm fiber lasers have low absorption coefficient of approximately 0.02 cm^−1^ in water, which results in less than 0.2% energy loss per millimeter of transmission, the beam partially penetrates the water layer before reaching the biofilm, leading to beam dispersion and refraction. This minimal attenuation enables efficient laser energy delivery to the submerged biofilm surface^25^. Higher laser power values may also contribute to thermal blooming effects, where the beam diameter expands due to localized heating of the surrounding water, resulting in wider removal regions but reduced energy density at the center. Additionally, at higher laser speeds, the reduced interaction time between the laser and biofilm limits heat transfer, preventing uniform width expansion. This explains why removal width does not consistently increase with laser speed, as the shortened exposure time counteracts the expected broadening effect.

**Fig. 16:**
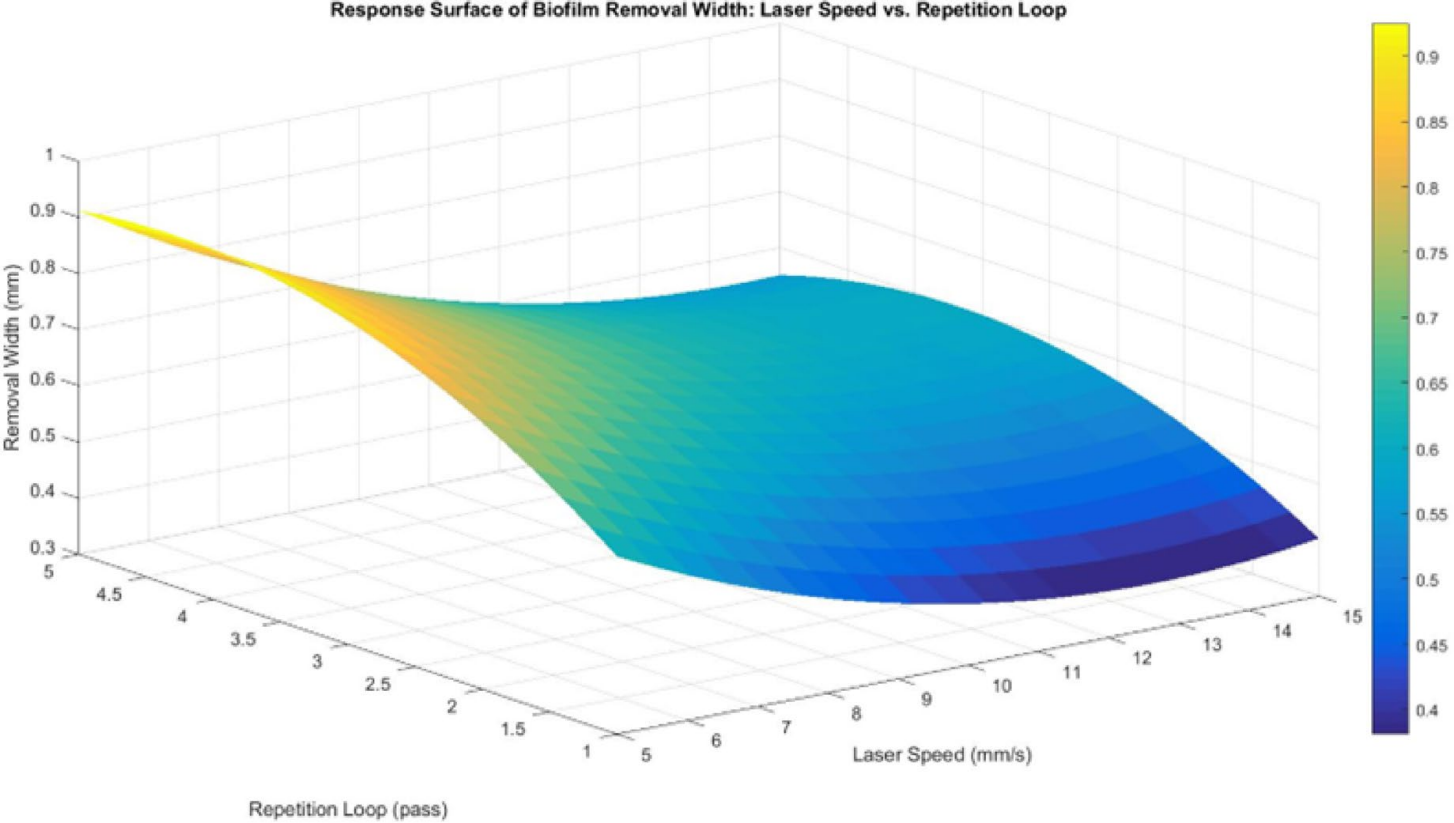
Response surface for biofilm removal width between laser speed and repetition loop.

The response surface between laser speed and repetition loop (Fig. 16) shows minimal variation in width across laser speed levels, supporting the lack of statistical significance for laser speed in the regression model. This suggests that laser speed does not strongly influence width, likely due to beam diffusion effects in water. Since the fiber laser interacts with both water and the biofilm, scattering dominates the process, preventing a clear correlation between laser speed and width expansion. Additionally, aluminium’s high thermal conductivity disperses heat away from the target area, reducing the likelihood of extended lateral ablation. As a result, changes in laser speed do not significantly impact removal width, as the heat dissipation effect counteracts the expected influence of laser speed variations.

The regression model indicates that laser power and repetition loop influence biofilm removal width in a nonlinear manner, while laser speed has no significant effect. This is further supported by the response surface plots, which show that removal width increases with increasing laser power. This trend is likely influenced by secondary factors such as water disturbance, microbubble formation, and localized heat flow in the aluminium substrate. A primary contributor to this widening effect is the Gaussian energy distribution of the laser beam, in which the intensity peaks at the center and gradually decreases toward the edges. As laser power increases, a larger portion of the beam exceeds the material-specific ablation threshold, effectively enlarging the ablation zone. This mechanism may explain why laser power has a more pronounced impact on removal width than repetition loops.

The highest predicted biofilm removal width of 1.275 mm was achieved at 90% laser power, 5 mm/s laser speed, and 5 repetition loops (Table 10). Similar to the biofilm removal depth prediction, this value was not experimentally validated due to inherent variability in biofilm growth conditions.

**Table 10:** Summary of the optimized laser parameters and predicted biofilm removal width.

Parameter	Optimized value	Predicted width (µm)	Note
Laser power (% of 50 W)	90	1.275	Based on fitted regression model
Laser speed (mm/s)	5		
Repetition loop	5		

In short, underwater laser ablation using a 1064 nm fiber laser effectively removed *C. crescentus* biofilm from aluminium substrates. Compared to conventional air-based processing, the submerged environment provided better thermal control, as the surrounding water absorbed excess heat and helped minimize thermal damage to the material. The presence of water also aided in cleaner removal by reducing debris accumulation and enhancing energy confinement through cavitation effects^27,28^. Although a direct comparison between underwater and air-based laser ablation could offer additional insight, but the evaluation is not feasible in this study due to distinct differences in beam interaction with the medium. In underwater settings, specifically using PYE nutrient broth, the laser beam experiences refraction at the interface caused by differing refractive indices, which results in a narrowed beam spot and increased surface intensity. It is noted that energy loss also occurs during transmission through liquid, estimated at approximately 0.2% per millimeter in clear water. The turbid nature of PYE further attenuates the laser energy, making its behaviour distinct from that in air. Additionally, air-based laser ablation would require an entirely different set of experimental conditions. Given these complexities and aforementioned explanation, the results from underwater laser processing cannot be directly compared to air-based outcomes.

Although underwater laser treatment has demonstrated effective results in targeted biofilm removal, several limitations remain. Variability in biofilm characteristics such as thickness, density, and adhesion strength, can influence the removal outcome and may require adjusted laser parameters for each condition. The method also relies on precise control of laser settings to ensure consistency, which may reduce its practicality in field applications. Preliminary testing is often necessary to determine suitable laser processing parameters based on the specific biofilm properties. Additionally, scaling up the method or applying it to complex surfaces may involve higher costs and present technical challenges. Future research should aim to address these limitations to enhance the method’s applicability and improve overall system efficiency.

## Conclusion

This study demonstrated the feasibility of fiber laser treatment as a method for *C. crescentus* biofilm removal from aluminium substrates submerged in 3 mm depth of PYE media. The BBD was used to analyze the effects of laser power, laser speed, and repetition loop in determining biofilm removal efficiency. The findings highlight that repetition loops play a pivotal role in determining removal depth efficiency, as multiple repetition loops help counteract energy losses due to water absorption and aluminium’s heat dissipation. While higher laser power enhances this effect by increasing energy delivery, its standalone influence remains limited. Contrary to expectations, laser speed did not significantly impact biofilm removal depth, likely due to thermal dispersion and water-induced energy dissipation, which prevent sustained energy accumulation. These results suggest that underwater laser processing requires repeated exposure to compensate for energy diffusion losses, emphasizing the need for an optimized multi-loop laser strategy to achieve effective and consistent biofilm removal. As for biofilm removal width, regression analysis showed that laser power and repetition loops influence biofilm removal efficiency in a non-linear manner, while laser speed had a minimal effect. Response surface plots further confirmed that the removal width expanded in a curved pattern with increasing laser power, likely due to secondary effects such as water-induced dispersion, microbubble formation, and aluminium’s thermal properties. These findings highlight the complexity of laser-material interactions in submerged environments, where beam scattering, water turbulence, and heat transfer dynamics must be considered to achieve optimal biofilm removal width. Future research should focus on controlling water turbulence and refining laser pulse dynamics to minimize energy dispersion, ensuring more consistent removal patterns. Additionally, exploring biofilm adhesion properties could provide further insights into how substrate conditions influence laser-biofilm interactions. Despite these complexities, this study reinforces the importance of non-linear effects in submerged laser processing and underscores the need for a carefully optimized laser strategy to enhance biofilm removal efficiency while minimizing unintended substrate modifications.

## Electronic supplementary material

Below is the link to the electronic supplementary material.

Supplementary Material 1

Supplementary Material 2

## Data Availability

Data are available in the manuscript and supplementary information files.
